# Overcoming Pavlovian bias in semantic space

**DOI:** 10.1038/s41598-021-82889-8

**Published:** 2021-02-09

**Authors:** Sam Ereira, Marine Pujol, Marc Guitart-Masip, Raymond J. Dolan, Zeb Kurth-Nelson

**Affiliations:** 1 Max Planck UCL Centre for Computational Psychiatry and Ageing Research, UCL, London, WC1B 5EH UK; 2 Wellcome Centre for Human Neuroimaging, UCL, London, WC1N 3BG UK; 3 Sorbonne Université, Paris, France; 4 Aging Research Centre, Karolinska Institute, 171 65 Stockholm, Sweden; 5 DeepMind, London, N1C 4AG UK

**Keywords:** Human behaviour, Decision

## Abstract

Action is invigorated in the presence of reward-predicting stimuli and inhibited in the presence of punishment-predicting stimuli. Although valuable as a heuristic, this Pavlovian bias can also lead to maladaptive behaviour and is implicated in addiction. Here we explore whether Pavlovian bias can be overcome through training. Across five experiments, we find that Pavlovian bias is resistant to unlearning under most task configurations. However, we demonstrate that when subjects engage in instrumental learning in a verbal semantic space, as opposed to a motoric space, not only do they exhibit the typical Pavlovian bias, but this Pavlovian bias diminishes with training. Our results suggest that learning within the semantic space is necessary, but not sufficient, for subjects to unlearn their Pavlovian bias, and that other task features, such as gamification and spaced stimulus presentation may also be necessary. In summary, we show that Pavlovian bias, whilst robust, is susceptible to change with experience, but only under specific environmental conditions.

## Introduction

Animals can learn which behaviours should be repeated to gain future rewards^1^. In addition to learning new behaviours via instrumental learning, animals also exhibit a Pavlovian bias whereby they tend to approach appetitive stimuli, even if greater rewards are available by counter-intuitively retreating. An early account of this Pavlovian bias involved an experiment in cockerel chicks, using an ingenious contraption^2^. Food was available on a motorised cart along a linear track, rigged with a sensor such that whenever the chick moved, the cart would move in the same direction along the track, but at twice the speed. Empirically, the animals could not learn the optimal behaviour of retreating from the food in order to obtain it.

This bias is widely considered to result from the way dopamine neurons signal information about rewards in the environment. On one hand, dopamine signals a reward prediction error, the discrepancy between the reward that was experienced and the reward that had been expected, which is used to drive learning^3–5^. On the other hand, dopamine neurons also directly modulate behaviour by invigorating motoric responses, particularly approach responses^6,7^. A leading theory posits that evolution selected these two separate functions to be performed by the same dopaminergic signalling pathway because the functions are very often correlated^1,8–10^. Indeed, the extent to which human behaviour is influenced by these biases can be modulated by targeting dopamine receptors^11–14^.

Pavlovian bias is proposed to contribute to maladaptive and risky behaviours such as drug-seeking^15,16^ and gambling^17^, and may contribute also to depression^18^. It would be of great interest to find behavioural interventions that could mitigate the bias when it is harmful. One recent study suggests that Pavlovian bias can change over time^19^, but to our knowledge it has never been attempted to reduce the bias through behavioural training.

Pavlovian influences on instrumental learning have historically been investigated using Pavlovian-Instrumental transfer tasks^20^, with explicit pavlovian and instrumental training phases. More recently however, Pavlovian influences have been measured using valenced go/no-go tasks^21^, where subjects are incentivised to engage in pure instrumental learning, but optimal choice behaviour is contaminated by an incidental Pavlovian bias. The task requires subjects to decide between making an active motoric response (‘go’) and withholding a motoric response (‘no-go’), in order to obtain a reward or avoid a loss. The cross product of these two conditions creates four types of trials: ‘go to win’, ‘go to avoid losing’, ‘no-go to win’, and ‘no-go to avoid losing’. Subjects consistently show two strong biases. The first bias is a ‘go’ bias, which leads people to more often choose ‘go’ than ‘no-go’, irrespective of whether the subject is playing a win or a loss condition. The second bias is a Pavlovian bias, an augmented disposition to emit a ‘go’ response to gain a reward and an attenuated disposition to act in the face of a potential loss^11,12,14,19,21–25^.

Here we trained subjects for 3 days on multiple variants of the valenced go/no-go task, to identify whether humans can learn to overcome their Pavlovian bias, and if so, what are the environmental conditions conducive to this unlearning process. One novel contribution of the current work is to introduce a valenced go/no-go task where the choice, rather than being in a motoric space, is in a ‘semantic space’^26–28^. Here the subject does not decide to emit or withhold a physical movement, but selects a verbal response with a meaning associated with movement or a response with a meaning associated with no movement. We expected that a Pavlovian bias would also be evident in this alternative version of the task. Were this the case, it would provide a new modality for behavioural tasks in assessing Pavlovian bias. One important difference of this semantic response domain is that it does not require participants to respond within a short time-window, as in the traditional motoric go/no-go task. We were interested to see whether subjects might be more or less susceptible to unlearning the Pavlovian bias in this semantic space.

In a series of five experiments, we show that subjects exhibit a strong Pavlovian bias in the semantic response domain in addition to the motoric response domain. Furthermore, we find that the bias is unaffected by training, with one important exception. In a variant of the task, where the semantic response domain is combined with gamification, relaxed time constraints and spaced stimulus presentation, an initially strong bias significantly reduces with training. Strikingly, after such training, these subjects also exhibited a reduced Pavlovian bias in an independent task. Our results suggest that instrumental learning with semantic response options is necessary but not sufficient to enable subjects to unlearn their Pavlovian biases.

## Results

**Figure 1:**
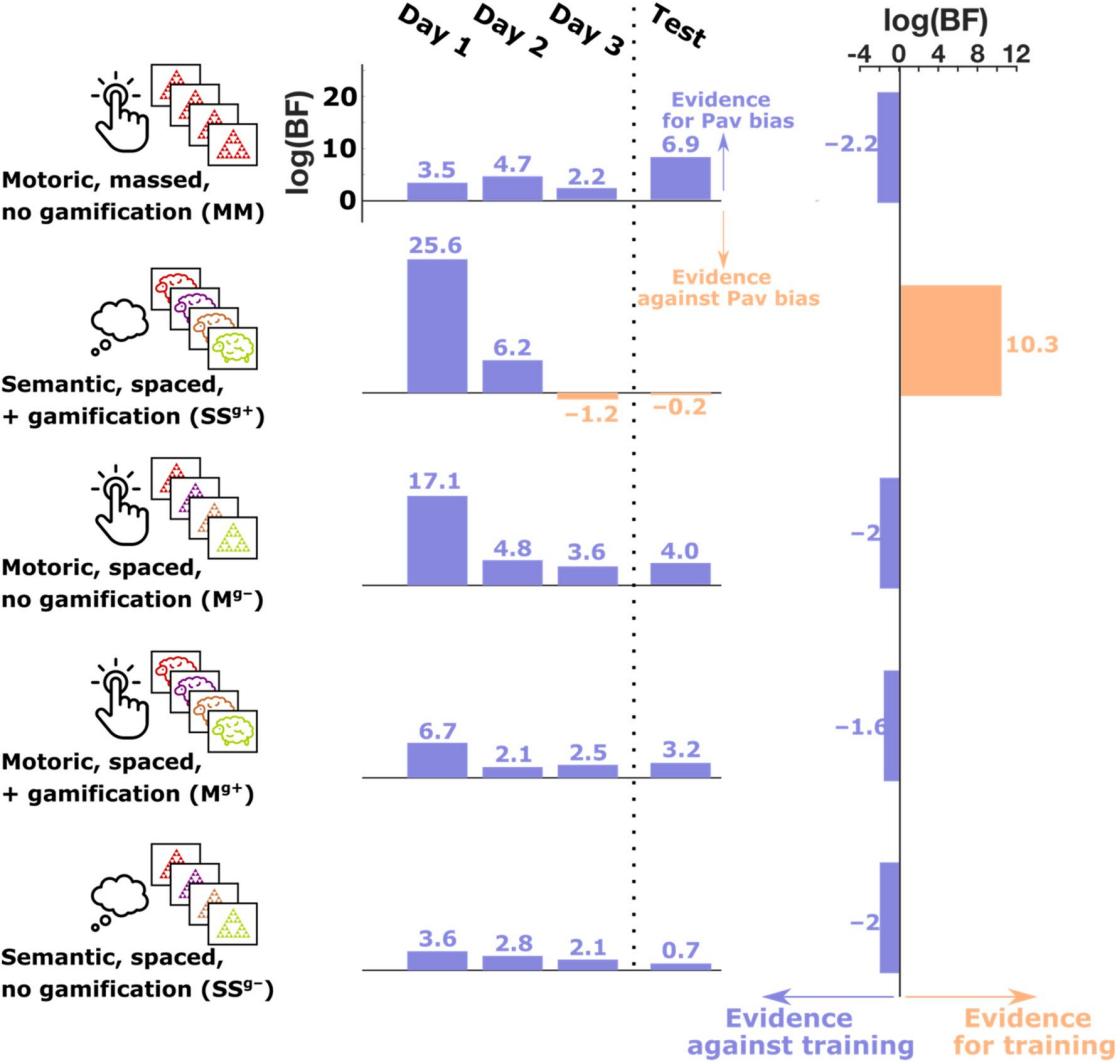
Summary of experiments and findings. Each of the five rows represents one of the five experiments. In each experiment, subjects played three training sessions on 3 consecutive days followed by a test session with a different task (the test task was SS^g+^ in experiment 1 and MM in all other experiments). The training task for each experiment varied in several features, including whether the task responses were ‘motoric’ or ‘semantic’, whether stimulus presentation was ‘massed’ or ‘spaced’ and whether or not the task was gamified. The middle column shows the evidence, on each training session, for or against a Pavlovian bias. This is the log Bayes factor, comparing a model that includes a two-way interaction term between choice and valence on accuracy, against a null model that excludes this interaction term (see “Methods”). The third column shows the evidence, in each experiment, for or against a training effect on Pavlovian bias across the three sessions. This is the log Bayes factor, comparing a model that includes a three-way interaction term between choice, valence and training session on accuracy, against a null model that excludes this interaction term (see “Methods”). Each training session showed substantial evidence in favour of a Pavlovian bias, except for the final training session in experiment 2, which showed substantial evidence against a Pavlovian bias. This experiment was unique in that the training task combined a semantic response domain with gamification and spaced stimulus presentation. Whilst other experiments showed a reduction in evidence for a Pavlovian bias over the three training sessions, it was only when these features were all combined in experiment 2, that we observed a true training effect.

We conducted five separate experiments. In each experiment, we trained subjects on a behavioural task for 3 days in a row and then on the 3rd day we tested them on a different behavioural task. Each experiment used a slightly different valenced go/no-go task for training. The core features of these tasks, and the main findings from each experiment are summarised in Fig. 1.

### Experiment 1: No training effect on a massed motoric go/no-go task

**Figure 2:**
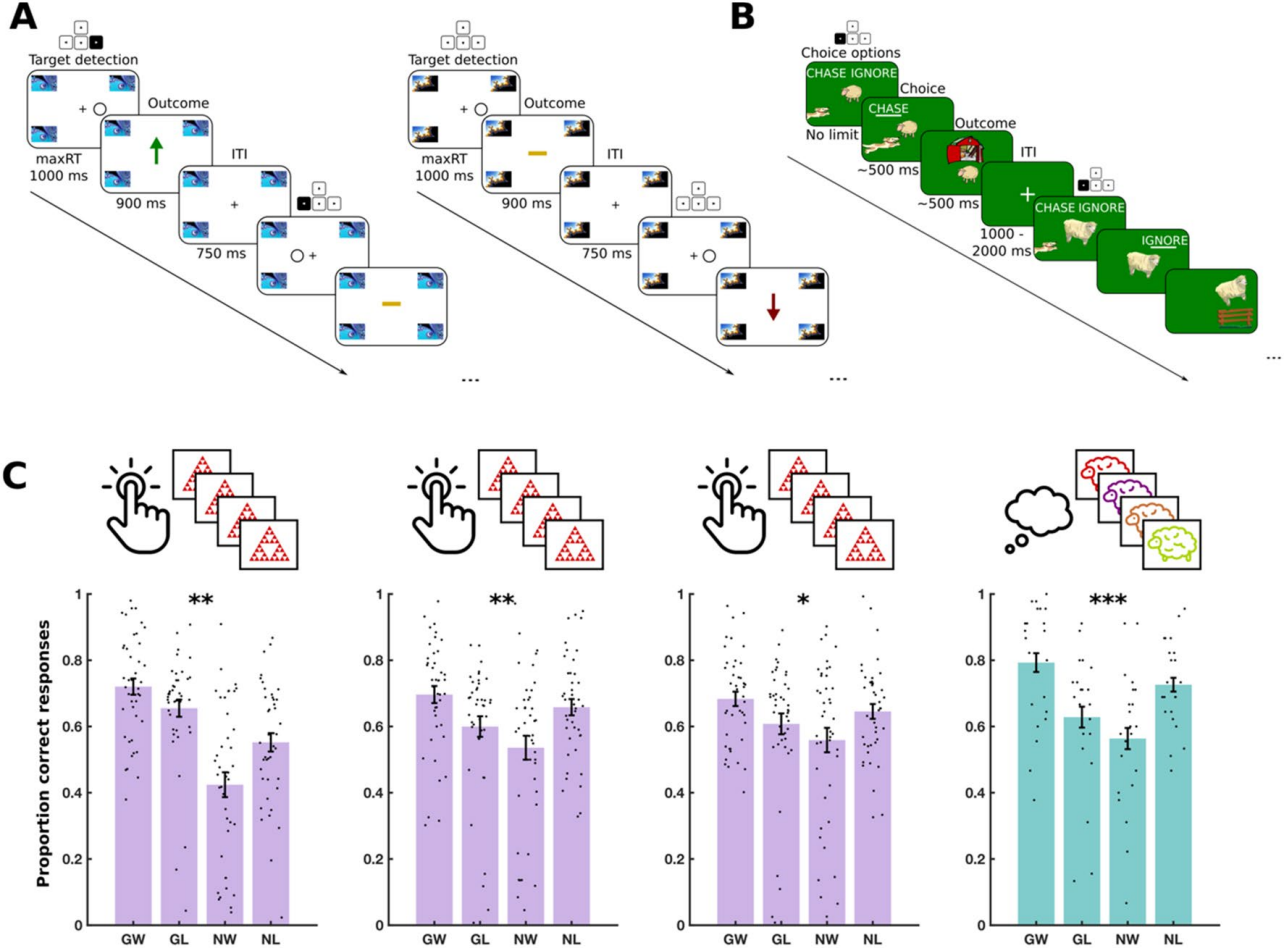
Experiment 1: Task design and results. (**A**) MM trial structure. Left: 2 example trials from a single mini-block in the ‘go to win’ condition. The highlighted buttons on keypads show what correct responses would be. In the first trial, the subject should have pressed the right arrow key, as indicated by the highlighted arrow key above the target detection frame. This yields positive feedback, as shown in the subsequent outcome frame. In the second trial, the target appears on the left and so the subject should have pressed the left arrow key. Due to the probabilistic nature of the feedback, this may result in a neutral outcome, as indicated by the yellow bar in the final outcome frame. Right: 2 example trials from a single mini-block in the ‘no-go to avoid losing’ condition. The subject should not press any keys. After the first trial, this yields neutral feedback. However, after the second trial, due to the probabilistic nature of the feedback, this yields a negative outcome, as indicated by the red arrow. (**B**) SS^g+^ trial structure. Two example trials are shown. The highlighted buttons on keypads show what correct responses would be. The first trial shows a sheep in the ‘go to win’ condition. The subject correctly chooses ‘chase’ and the sheep is successfully herded into the barn in the outcome phase (positive feedback). The second trial shows a sheep in the ‘go to avoid losing’ condition. The subject should choose ‘chase’ but incorrectly choses ‘ignore’ and the sheep is shown escaping the barn (negative feedback). (**C**) Performance in three daily training sessions of MM and a fourth testing session of SS^g+^ on day 3. Each dot shows the average performance for an individual subject in one of the four conditions: Go to win (GW), go to avoid losing (GA), no-go to win (NW) and no-go to avoid losing (NL). Error bars show standard error of the mean across participants. Significance stars show p-value ranges for the interaction between valence (win/avoid losing) and choice (go/no-go) on performance. *P < 0.05. **P < 0.01. ***P < 0.001.

We trained 42 subjects on a ‘massed motoric go/no-go task’ (MM) (Fig. 2A). In this paradigm, subjects used trial-and-error to learn which of four possible conditions a novel abstract stimulus represented. In the ‘go to win’ condition, subjects had to elicit a motoric response (button press) to win money. In the ‘go to avoid losing’ condition, subjects had to withhold a motoric response (no button press) to avoid losing money. In the ‘no-go to win’ condition, subjects had to withhold a motoric response to win money. Finally, in the ‘no-go to avoid losing’ condition, subjects had to withhold a motoric response to avoid losing money. Choices yielded probabilistic outcomes (see “Methods”).

Subjects played the game 3 days in a row, each day encountering novel stimuli. The task consisted of a series of miniblocks with 10–15 ‘massed’ presentations of the same stimulus in a row. Each miniblock, on each day, was associated with its own unique stimulus. This meant that it was not possible for subjects to infer the condition of one stimulus from experience with previous stimuli.

A subset of 22 subjects played an additional ‘spaced semantic go/no-go task with gamification’ (SS^g+^) on day 3 (Fig. 2B). We included this task, to assess whether any training on MM might transfer to an unseen task. This task differed from MM in several ways. Firstly, it presented subjects with a detailed cover story whereby they played the role of a sheepdog herding sheep (see “Methods”). Rather than pressing or not pressing a button, subjects were instead required to choose a verbal response ‘chase’ or ‘ignore’. This enabled us to test for Pavlovian bias in a semantic response domain rather than a motoric response domain. The same motoric button press was required to elicit either of these two responses. Secondly, the stimuli were not abstract patterns, but consisted animated cartoon sheep with unique distinguishing features. Thirdly, stimuli were presented in a spaced fashion (interspersed) rather than in a massed fashion. The task consisted of independent blocks and, within a block, subjects encountered four different stimuli repeatedly, one for each of the four conditions. Each block contained its own unique set of four stimuli.

We computed accuracy within each of the four conditions, separately, as the proportion of correct responses (Fig. 2C). For instance, in ‘go to win’ trials, accuracy was the proportion of trial with which the subject chose ‘go’. A Pavlovian bias influences people to ‘go’ in response to rewarding stimuli and to ‘no go’ in response to punishing stimuli. This results in high performance on ‘go to win’ trials and ‘no go to avoid losing’ trials, in comparison to ‘go to avoid losing’ and ‘no go to win’ trials. We therefore quantified Pavlovian bias as the effect of a two-way interaction between choice (go or no-go) and valence (win or avoid losing) on accuracy, in a mixed logistic regression model, fit to data from all three training sessions (see “Methods”).

In this model, we observed a significant main effect of valence on accuracy [F(1, 68,972) = 12.48, P < 0.001] with higher accuracy on ‘avoid losing’ trials than ‘win’ trials. There was also a significant main effect of training session on accuracy [F(1, 68,972) = 6.98, P < 0.001] with overall accuracy higher on days 2 and 3 than on day 1. We found a significant two-way interaction between choice and valence on accuracy [F(1, 68,972) = 10.3, P = 0.001] reflecting an overall Pavlovian bias. Supplementary Table 1 shows the full table of effects.

Interestingly, there was no three-way interaction between choice, valence and session on accuracy [F(2, 68,972) = 0.33, P = 0.72], providing no evidence for a change in Pavlovian bias across the three training sessions. By computing Bayes factors, from a Bayesian repeated measures ANOVA (see “Methods”), we found substantial evidence in favour of the null hypothesis that there was no three-way interaction, with respect to the alternative hypothesis that there was a three-way interaction [log(BF) = 2.22]. However, we did observe a significant two-way interaction between choice and session on accuracy [F(2, 68,972) = 6.4, P = 0.002], representing a significant reduction in subjects’ overall propensity to choose a ‘go’ response as they proceeded through the three training sessions. These results show that over the 3 days of training, subjects learned to overcome a ‘go’ bias, and therefore score more points, whilst a Pavlovian bias persisted.

We also fit mixed regression models to each individual training session, and found a significant two-way interaction effect between choice and valence on accuracy in every session [session 1: F(1, 23,096) = 9.99, P = 0.002, session 2: F(1, 4676) = 7.53, P = 0.006, session 3: F(1, 4676) = 4.99, P = 0.025]. In other words, there was evidence for a Pavlovian bias in each and every individual training session. The Pavlovian bias was also detectable in the testing session on SS^g+^, as a significant two-way interaction between choice and valence on accuracy [F(1, 3956) = 14, P < 0.001].

### Experiment 2: Successful training on a spaced semantic go/no-go task

**Figure 3:**
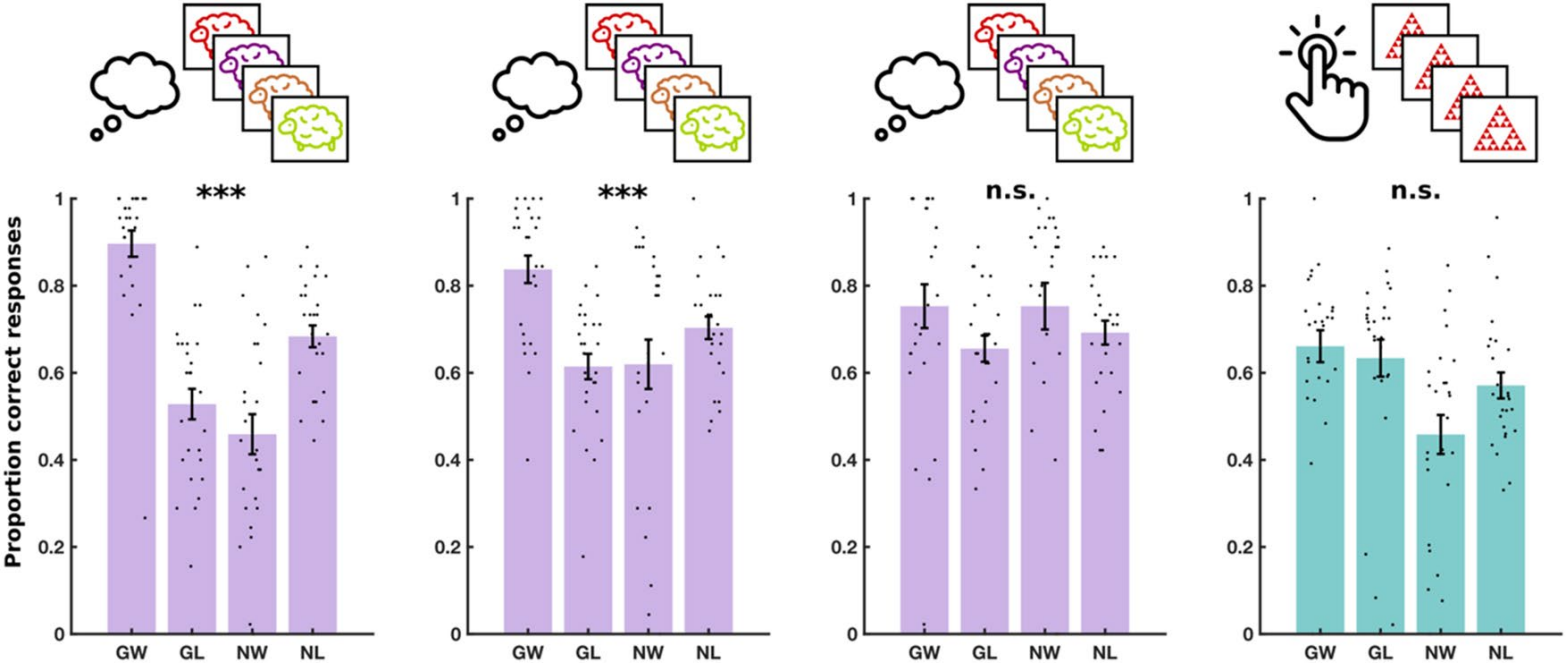
Experiment 2 results. Performance in three daily training sessions of SS^g+^ and a fourth testing session of MM on day 3. Each dot shows the average performance for an individual subject in one of the four conditions: Go to win (GW), go to avoid losing (GA), no-go to win (NW) and no-go to avoid losing (NL). Error bars show standard error of the mean across participants. Significance stars show P-value ranges for the interaction between valence (win/avoid losing) and choice (go/no-go) on performance. ***P < 0.001. n.s. P > 0.05.

Here we simply reversed the training and testing tasks from experiment 1. We trained a new sample of 26 subjects on SS^g+^ for 3 days. On the 3rd day we also tested them on MM. We conducted the same analysis as for experiment 1 (Fig. 3). This experiment was initially run with a sample of 14 subjects. After analysing the data, we replicated the experiment with an additional sample of 12 subjects, where we replicated the effects seen in the original sample. However, due to the small sample sizes, we report these data by pooling the two datasets into a single analysis of 26 subjects.

As in experiment 1, there was a significant two-way interaction between choice and valence on accuracy [F(1, 14,028) = 43.6, P < 0.001], indicating an overall Pavlovian bias. However, we did observe a significant three-way interaction between choice, valence and session on accuracy [F(2, 14,028) = 10.08, P < 0.001] indicating a significant change in Pavlovian bias over the 3 training days. Supplementary Table 2 shows the full table of effects. Separate analyses on each individual training session found that whilst a two-way interaction between valence and choice was present on day 1 [F(1, 4676) = 45.29, P < 0.001] and day 2 [F(1, 4676) = 14.26, P < 0.001], it was entirely absent on day 3 [F(1, 4676) = 1.03, P = 0.31]. Furthermore, a Bayesian repeated measures ANOVA indicated substantial evidence in favour of the null hypothesis that there was indeed no Pavlovian bias on the third training session [log(BF) = 1.24].

We noted that the Pavlovian bias on day 1 was particularly strong (Figs. 1 and 3, [log(BF) = 25.6]). To ensure that the training effect was not simply an artefact, due to day 1 being an outlier with an unusually strong Pavlovian bias, we repeated the logistic regression, excluding day 1 from the analysis. On this ‘restricted’ dataset we still observed a significant three-way interaction between choice, valence and session on accuracy [F(1, 9352) = 4.7, P = 0.03], indicating a significant reduction in Pavlovian bias from day 2 to day 3, This suggests that the observed reduction in Pavlovian bias across the three training sessions was not merely due to an unusually strong starting Pavlovian bias on day 1.

These results support an interpretation that, over 3 days of training on SS^g+^, subjects learned to overcome their Pavlovian bias. Interestingly, there was no main effect of session on accuracy [F(2, 1408) = 0.34, P = 0.71], indicating that overall performance did not improve, or deteriorate, as subjects proceeded through the three training sessions. Rather, subjects showed a selective improvement on Pavlovian-incongruent trials, and selective deterioration on Pavlovian-congruent trials. Furthermore, in the MM testing session on day 3, there was no two-way interaction between valence and choice on accuracy [F(1, 13,802) = 2.02, P = 0.15], providing no evidence for a Pavlovian bias in the independent transfer task. This suggests that the Pavlovian training that subjects experienced in SS^g+^ was transferable to an unseen task. More specifically, it was transferable from a semantic to a motoric space. However, using a Bayesian repeated measures ANOVA, we found that the evidence in favour of the null hypothesis, of no Pavlovian bias on MM, was only weak [log(BF) = 0.21].

In summary, this experiment found substantial evidence that subjects could learn to overcome their Pavlovian bias in SS^g+^, with weak evidence that this training effect transferred to a novel task.

### Experiment 3: Spaced stimulus presentation is not sufficient for training

**Figure 4:**
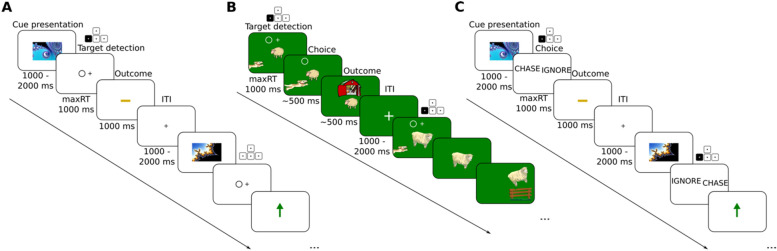
Task design in experiments 3, 4 and 5. (**A**) Task design for M^g−^. Two example trials are shown. The highlighted buttons on keypads show what correct responses would be. The first trial shows a stimulus in the ‘go to avoid losing’ condition. The subject correctly presses and neutral feedback is shown in the outcome phase. The second trial shows a stimulus in the ‘no go to win’ condition. The subject correctly chooses to not press and positive feedback is shown in the outcome phase. (**B**) Task design for M^g+^. Two example trials are shown. The highlighted buttons on keypads show what correct responses would be. The first trial shows a stimulus in the ‘go to win’ condition. The subject correctly presses and positive feedback is shown in the outcome phase. The second trial shows a stimulus in the ‘go to avoid losing’ condition. The subject correctly presses, but due to the probabilistic nature of the feedback, negative feedback is shown in the outcome phase. (**C**) Task design for SS^g−^. Two example trials are shown. The highlighted buttons on keypads show what correct responses would be. The first trial shows a stimulus in the ‘go to avoid losing’ condition. The subject correctly chooses ‘chase’ and neutral feedback is shown in the outcome phase. The second trial shows a stimulus in the ‘no go to win condition. The subject correctly chooses ‘ignore’, and positive feedback is shown in the outcome phase.

There were several differences between SS^g+^ and MM and so it was not clear which features might lead to successful training in one task (experiment 2) but not in the other (experiment 1). To try and isolate features necessary and sufficient for training away a Pavlovian bias, we devised several more experiments. In experiment 3, we assessed whether spaced stimulus presentation was sufficient to enable subjects to overcome their Pavlovian biases. To this end, we trained a new sample of 20 subjects on a spaced motoric go/no-go task without gamification (M^g−^) (Fig. 4A). This task was structurally identical to SS^g+^ but had no cover story, used abstract fractal stimuli instead of sheep, and required responses in the motoric rather than the semantic domain. On the 3rd day, subjects were also tested on MM.

**Figure 5:**
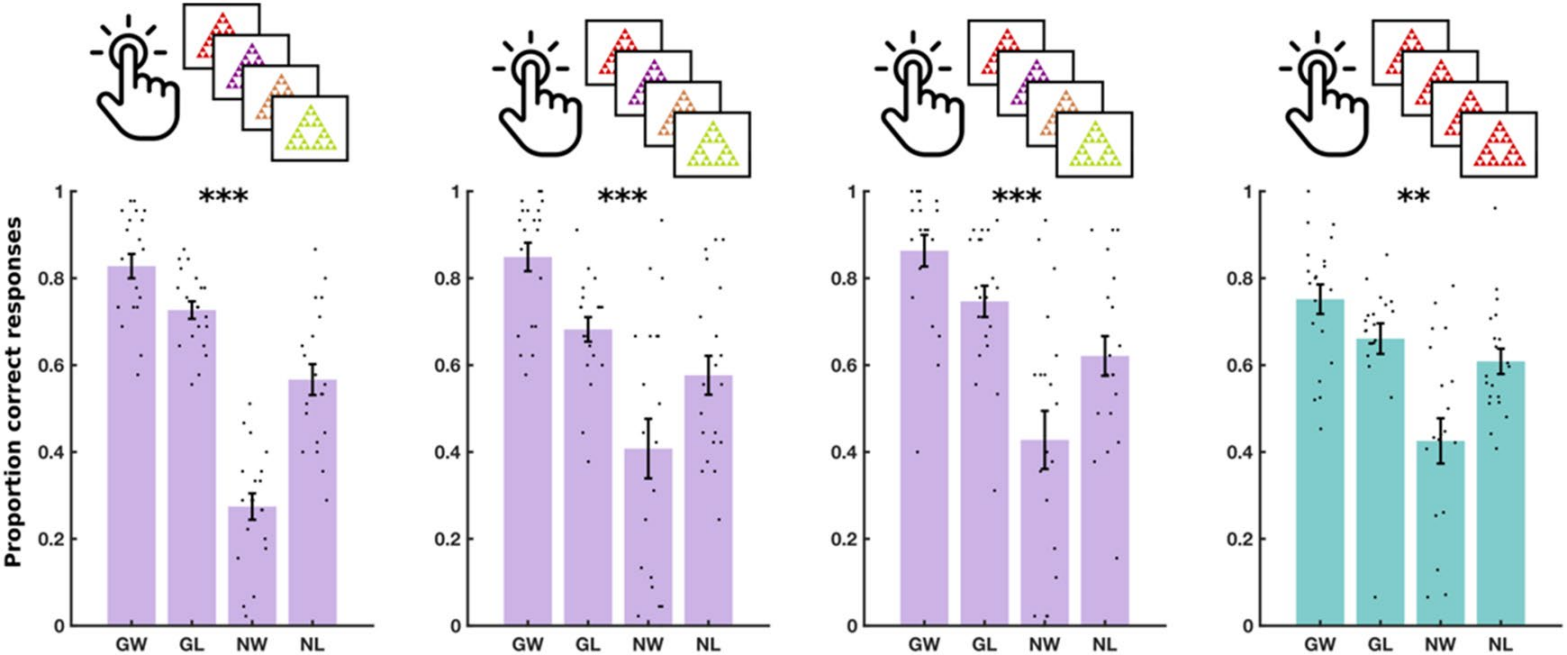
Experiment 3 results. Performance in three daily training sessions of M^g−^ and a fourth testing session of MM on day 3. Each dot shows the average performance for an individual subject in one of the four conditions: Go to win (GW), go to avoid losing (GA), no-go to win (NW) and no-go to avoid losing (NL). Error bars show standard error of the mean across participants. Significance stars show p-value ranges for the interaction between valence (win/avoid losing) and choice (go/no-go) on performance. **p < 0.01. ***p < 0.001.

The results for the M^g−^ training experiment are shown in Fig. 5 and Supplementary Table 3. We observed a significant two-way interaction between choice and valence on accuracy [F(1, 10,788) = 31.72, P < 0.001], indicating again an overall Pavlovian bias. There was no three-way interaction between choice, valence and session on accuracy [F(2, 10,788) = 0.11, P = 0.89], indicating no evidence of a training effect. Furthermore, a Bayesian repeated measures ANOVA indicated substantial evidence favouring the null hypothesis of no training effect [log(BF) = 1.96].

We also fit mixed regression models to each individual training session, and found a significant two-way interaction effect between choice and valence on accuracy in every session [session 1: F(1, 3596) = 33.26, P < 0.001, session 2: F(1, 3596) = 17.9, P < 0.001, session 3: F(1, 3596) = 12.9, P < 0.001]. In other words, there was evidence for a Pavlovian bias in every individual training session. The Pavlovian bias was also detectable in the testing session on MM, as a significant two-way interaction between choice and valence on accuracy [F(1, 10,616) = 7.66, P = 0.006]. Thus, we failed to replicate the training effect that we observed with SS^g+^, suggesting that spaced stimulus presentation does not, on its own, explain why training was successful in experiment 2.

### Experiment 4: Gamification is not sufficient for training

In experiment 4, we assessed whether the gamified nature of SS^g+^ (cover story, familiar stimuli and engaging animations), was sufficient to enable subjects to overcome their Pavlovian biases. To this end, we trained a new sample of 18 subjects on a spaced motoric go/no-go task with gamification (M^g+^) (Fig. 4B). This task was structurally identical to SS^g+^ in experiment 2, except it assessed responses in the motoric domain, requiring subjects to decide to emit or withhold a button press in order to ‘chase’ or ‘ignore’ a sheep. On the 3rd day, subjects were also tested on MM.

**Figure 6:**
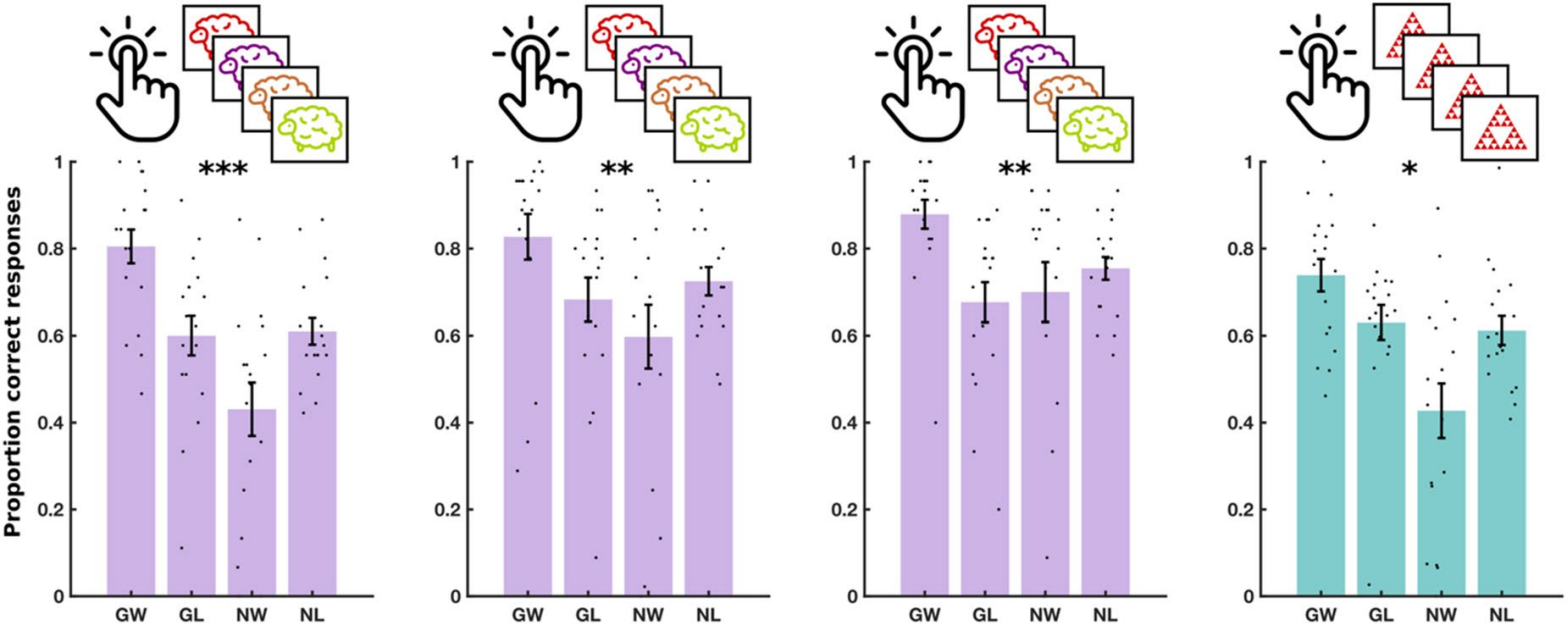
Experiment 4 results. Performance in three daily training sessions of M^g+^ and a fourth testing session of MM on day 3. Each dot shows the average performance for an individual subject in one of the four conditions: Go to win (GW), go to avoid losing (GA), no-go to win (NW) and no-go to avoid losing (NL). Error bars show standard error of the mean across participants. Significance stars show p-value ranges for the interaction between valence (win/avoid losing) and choice (go/no-go) on performance. *P < 0.05. **P < 0.01. ***P < 0.001.

The results for the M^g+^ training experiment are shown in Fig. 6 and Supplementary Table 4. Our analysis showed a significant two-way interaction between choice and valence on accuracy [F(1, 9708) = 12.1, P < 0.001], indicating an overall Pavlovian bias. However, there was no three-way interaction between choice, valence and session on accuracy [F(2, 9708) = 0.349, P = 0.71], suggesting again the absence of a training effect. This was established with a Bayesian repeated measures ANOVA, which indicated substantial evidence in favour of the null hypothesis of no training effect [log(BF) = 1.56].

We also fit mixed regression models to each individual training session, and found a significant two-way interaction effect between choice and valence on accuracy in every session [session 1: F(1, 3236) = 14.06, P < 0.001, session 2: F(1, 3236) = 9.5, P = 0.002, session 3: F(1, 3236) = 7.34, P = 0.007]. In other words, there was evidence for a Pavlovian bias in every individual training session. This Pavlovian bias was also detectable in the testing session on MM, as a significant two-way interaction between choice and valence on accuracy [F(1, 9554) = 6.46, P = 0.01].

As in experiment 3, we failed to replicate the training effect that we observed with SS^g+^. This suggests, firstly that gamification is insufficient for subjects to overcome their Pavlovian biases, and secondly, that the semantic response feature of SS^g+^ (which was absent from M^g+^) was necessary for the observed training in experiment 2.

### Experiment 5: Semantic response domain is not sufficient for training

Our results from the previous experiments suggested that providing responses within the semantic domain, rather than the motoric domain, was necessary for participants to learn to overcome their Pavlovian biases. We next tested whether this feature was sufficient. To this end, we trained a new sample of 20 subjects on a spaced semantic go/no-go task without gamification (SS^g−^) (Fig. 4C). This task was structurally identical to M^g−^ in experiment 3, but now assessed responses in the semantic domain, by using the cover story from SS^g+^, and instructing participants that each abstract stimulus represented a different sheep to ‘chase’ or ‘ignore’. On the 3rd day, subjects were also tested on MM.

**Figure 7:**
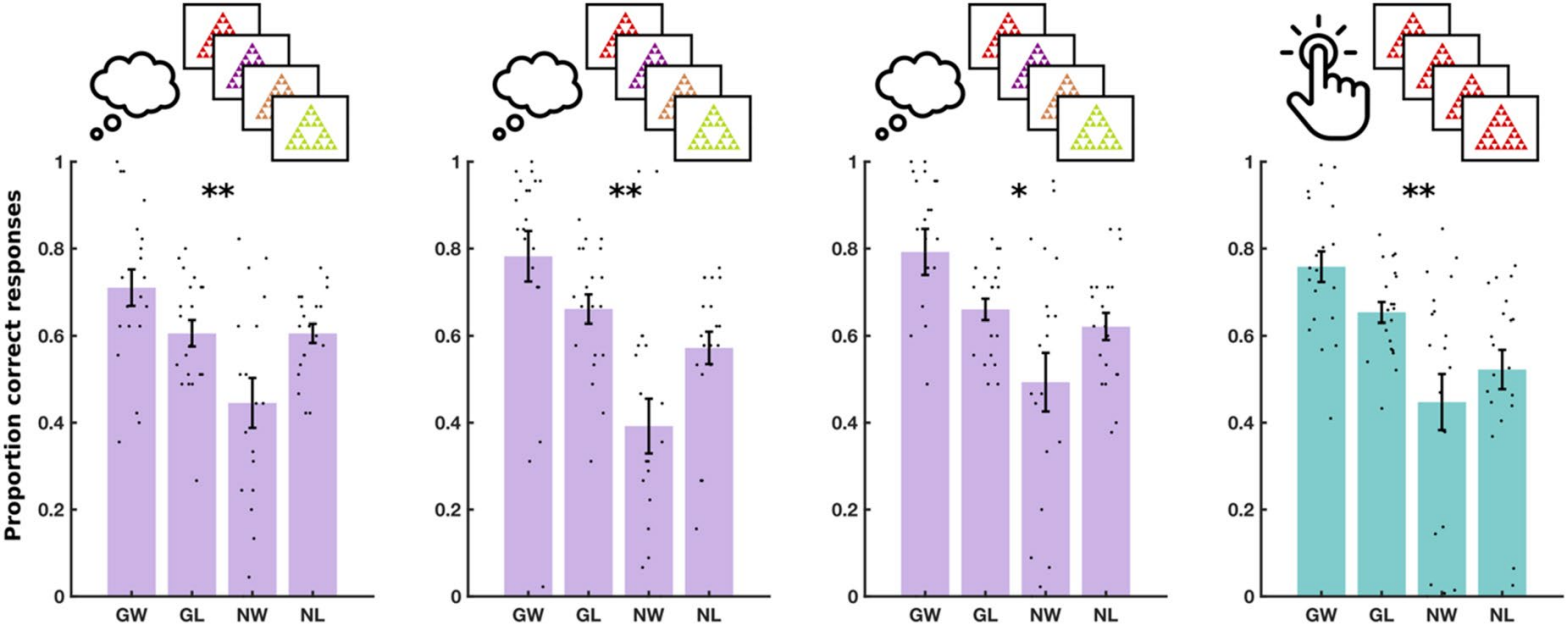
Experiment 5 results. Performance in three daily training sessions of SS^g−^ and a fourth testing session of MM on day 3. Each dot shows the average performance for an individual subject in one of the four conditions: Go to win (GW), go to avoid losing (GA), no-go to win (NW) and no-go to avoid losing (NL). Error bars show standard error of the mean across participants. Significance stars show P-value ranges for the interaction between valence (win/avoid losing) and choice (go/no-go) on performance. *P < 0.05. **P < 0.01.

The results for the SS^g−^ training experiment are shown in Fig. 7 and Supplementary Table 5. We detected a significant two-way interaction between valence and choice on accuracy [F(1, 107,880) = 9.24, P = 0.002], but no three-way interaction between valence, choice and session on accuracy [F(2, 10,788) = 0.43, P = 0.65]. Furthermore, a Bayesian repeated measures ANOVA showed substantial evidence in favour of the null hypothesis, that there was no training effect [log(BF) = 1.97]. The results indicate that a significant Pavlovian bias persisted throughout the three training sessions.

Again, we fit mixed models to each individual training session, and found a significant two-way interaction between choice and valence on accuracy in every session [session 1: F(1, 3596) = 9.46, P = 0.002, session 2: F(1, 3596) = 9.26, P = 0.002, session 3: F(1, 3596) = 6.12, P = 0.014]. In other words, there was evidence for a Pavlovian bias in every individual training session. The Pavlovian bias was also detectable in the testing session on MM, as a significant two-way interaction between choice and valence on accuracy [F(1, 10,616) = 8.05, P = 0.005].

Again, we failed to replicate the training effect that we observed with SS^g+^. This suggests that responding in the semantic domain is not sufficient to learn to overcome Pavlovian biases, and that other features of SS^g+^, such as the nature of the stimuli, animations or response timing, were necessary, along with the semantic responses.

### A common Pavlovian bias underpinning motoric and semantic responses

In experiments 1, 2 and 5, we consistently observed a form of Pavlovian bias which, as far as we know, has not been previously reported. We describe this as a Pavlovian bias in the ‘semantic’ domain. We replicated this finding in two different tasks, one using animated stimuli in a gamified task (experiments 1 and 2) and one using abstract stimuli in a non-gamified task, structured identically to a well-established valenced go/no-go task (experiment 5).

**Figure 8:**
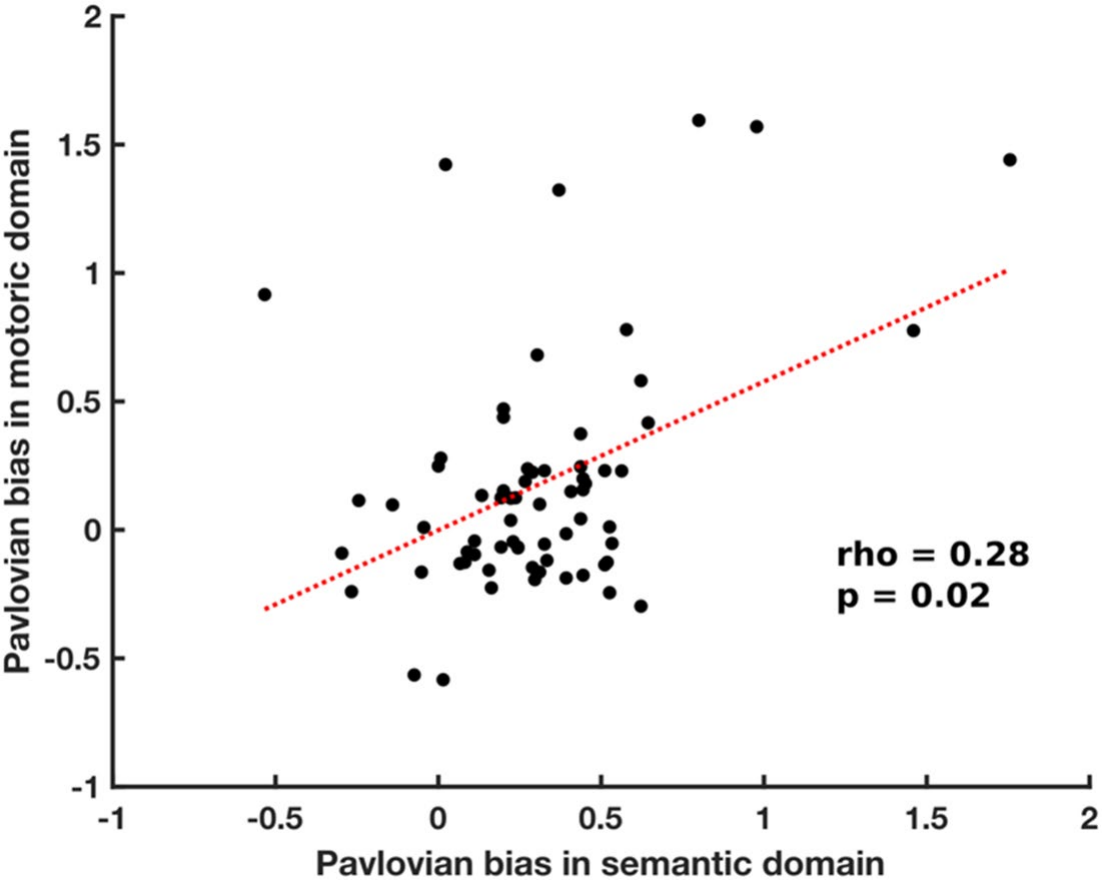
Correlation between Pavlovian bias in motoric and semantic domains. Each dot represents an individual subject. Data was pooled from experiments 1, 2 and 5. For each subject, a Pavlovian bias was computed in the motoric domain and in the semantic domain. These two Pavlovian biases are plotted against each other.

In these experiments, all subjects were trained for 3 days on a semantic go/no-go task and then tested on a motoric go/no-go task on day 3. We therefore obtained a measure of Pavlovian bias in both response domains. In order to assess whether these two behaviours express the same underlying computational bias, we pooled data from these three experiments and tested for a between-subject correlation in the bias between the two response domains. We calculated the interaction between valence and choice on accuracy, for each subject, by summing accuracy in the ‘go to win’ and ‘no go to avoid losing’ conditions, and then subtracting accuracy in the ‘go to avoid losing’ and ‘no go to win’ conditions. We averaged this value across the three training sessions to obtain a single value for the semantic domain. Crucially, we observed a significant correlation (Fig. 8) between the interaction value in the semantic domain and the equivalent value in the motoric domain [Spearman’s rho = 0.28, P = 0.02]. This supports the idea that the Pavlovian bias we observed in the semantic response domain is an expression of the same heuristic that accounts for the well-established bias in the motoric domain.

## Discussion

In this series of experiments, we trained subjects to engage in goal-directed, instrumental, learning and suppress their Pavlovian biases. We found that repeatedly exposing subjects to an instrumental learning task, for 3 days, was insufficient for subjects to overcome this bias. A notable exception to this was seen with SS^g+^ training, where subjects’ Pavlovian bias significantly diminished over 3 days of training, and the same subjects showed no evidence of Pavlovian bias in a distinct testing task, suggesting this training effect may have transferred to a new environment. In follow-up experiments we endeavoured to tease apart the features of SS^g+^ which might have been necessary, and sufficient, for this observed training effect.

An important feature of SS^g+^ was that responses were made in a semantic domain, rather than a motoric domain. Pavlovian behaviour is thought to be under the regulation of neuromodulatory signals, which multiplex value with vigour at choice^13,20,21,29,30^, with dopamine invigorating action in response to reward-associated cues, and serotonin perhaps inhibiting action in response to punishment-associated cues^31,32^. Pavlovian bias has conventionally been associated with physical action invigoration and inhibition, but has also been implicated in non-motoric decisions with more abstract choice modalities. For instance, framing effects on decisions between safe and risky options are also dopamine-dependent and can be explained by a model that incorporates a Pavlovian approach-avoid parameter^17,33,34^. Nevertheless, it has not been directly shown before that Pavlovian biases drive behaviour at a conceptual level of abstraction in addition to an action-level. Our findings support the notion that Pavlovian control extends beyond the action-level and can influence more abstract representations, in this case biasing the choice of a word which has semantic associations with behavioural activation or inhibition.

We found that the semantic response domain was necessary, but not sufficient, for successful training in SS^g+^. In experiment 4, subjects failed to overcome their Pavlovian biases in a task that was identical except for being in the motoric domain. Furthermore, the extent to which subjects showed a Pavlovian bias in the semantic domain was tightly linked to the expression of bias in the motoric domain. These two biases were correlated, and training away the Pavlovian bias in the semantic domain appeared to transfer to the motoric domain. Whilst our Bayesian analysis showed that the evidence for this transfer was only weak, it is notable that across five different groups of subjects who were tested on MM, this was the sole group who showed no evidence for expression of a Pavlovian bias.

There are several possible reasons why learning within the semantic response domain might facilitate training in this task. Firstly, semantic categorisation is well known to facilitate verbal episodic memory^35–39^. This is a learning technique whereby the learner looks for a latent semantic structure to cluster different items in a list of words. One possibility is that, in SS^g+^, subjects use semantic categorisation to facilitate the learning of individual stimuli. In other words, explicitly labelling a stimulus as ‘something I need to chase’ or ‘something I need to ignore’ might help subjects to recall the relevant condition for each stimulus, and acquire a generic model of the latent task structure. Secondly, semantic memories are known to be encoded in multimodal information processing brain regions^40,41^, and multimodal learning is faster than unimodal learning^42,43^. For instance, it is easier to recall visual stimuli if they were paired with distinct auditory stimuli at the time of learning. Indeed, it has been shown that humans more easily infer latent associative structure in a learning task when associations are between auditory states and visual states, as opposed to associations between states within a single modality^44^. In our study, learning using multimodal semantic representations may expedite the learning required to overcome Pavlovian biases. However, we also observed that the semantic response domain was not sufficient for subjects to overcome their Pavlovian biases, as demonstrated in experiment 5. There are therefore other features of SS^g+^ that were necessary for this learning to take place.

One of these features may pertain to the kinds of stimuli that we used in SS^g+^. In experiment 5, we retained the cover story and semantic domain but made the task simpler. We removed the animated animal stimuli and used abstract fractal stimuli and imposed a time constraint at the choice phase, changes that were sufficient to eliminate training effects that might have been facilitated by the semantic domain. Words representing animals, and animate objects, are better remembered than words representing inanimate objects^45–47^, while animacy also enhances learning about novel non-words^48,49^. In our study, using animated animal stimuli may have facilitated the learning of individual conditions. Interestingly, a recent study showed that animacy facilitates semantic categorisation^50^, and this kind of interaction might explain why we observed a training effect only when animate stimuli and a semantic response domain were combined. The combination of these features in SS^g+^ may have enabled subjects to represent the different conditions more explicitly than in other versions of the valenced go/no-go task. We suggest that future experiments might usefully probe how well subjects can report their knowledge of task structure in different versions of the valenced go/no-go task.

It is important to note that SS^g+^ was the only one of our tasks that did not impose a time constraint at the response phase. It is reasonable to ask whether the mere absence of time constraints is sufficient for the training effect that we observed. This is possible, but time constraints can only be removed when using the semantic response domain, as opposed to the motoric response domain. This would also, therefore, lead us to conclude that the semantic response domain was necessary for the training effect. Nevertheless, it may be the case that the semantic response domain only enables training, when coupled with relaxed time constraints. Follow-up work can easily test this hypothesis by conducting variants on these experiments, such as a version of SS^g+^ with time constraints imposed, or alternatively a version of SS^g−^ with time constraints removed.

We also cannot rule out a possible necessity of spaced learning. One important difference between MM and SS^g+^ was that the former used massed stimulus presentation, whilst SS^g+^ used spaced stimulus presentation. Reinforcement learning is facilitated when stimuli are encountered intermittently, with temporal spacing^51–53^. The lack of training effects in experiments 3, 4 and 5 demonstrate that spaced stimulus presentation is certainly not sufficient for training. In our experiments we did not test the efficacy of training on a task equivalent to SS^g+^ but with massed stimulus presentation, so we cannot say how important this feature is. Future experiments will be needed to determine whether or not spaced stimulus presentation is necessary for subjects to overcome their Pavlovian biases.

Whilst these behavioural biases are partially driven by a Pavlovian control system, there is also a contribution from an instrumental learning bias^12,13^. Whereas a Pavlovian bias is an effect at the choice phase, an instrumental bias introduces an asymmetric weighting of positive and negative reward prediction errors at outcome processing. In our experiments we cannot disentangle how these biases evolved over the course of the 3 days in the successfully trained SS^g+^ task, and consequently an avenue for future work will be to determine which environmental features enable subjects to adapt to both these biases, independently.

The results from these experiments have several limitations. Because we have not tested every possible task that can be generated by varying the response domain (motoric or semantic), gamification and temporal dynamics of stimulus presentation, we are unable to determine conclusively which task features are necessary and sufficient for Pavlovian training. Furthermore, it is likely that the space of task parameters that impact on this unlearning process is far larger than what we have investigated in the current study. Nevertheless, we hope that these results will help investigators to develop new paradigms and extend the task space in a systematic fashion. We also note that initial behaviour is not identical across the five different tasks that we used, which limits the extent to which we can directly compare behaviour across these tasks. It is reassuring that every task exposed a clear Pavlovian bias, allowing us to compare the dynamics of this specific behavioural feature throughout training. However, as the tasks are so different in other ways, we are restricted in the behavioural comparisons that can be made.

More generally, scepticism is warranted when relying on cross-experimental comparisons of the presence and absence of various effects. Whilst we have tried to mitigate this issue by computing positive Bayesian evidence for null effects, we believe the preliminary work presented here ought to be replicated in larger studies. Furthermore, ideally, one would investigate the impact of various task features on Pavlovian training, on the same sample of participants at different points in time. Although outside the scope of the current study, this could be an important line of future research, that we hope can be built on the results presented here.

In summary, our experiments demonstrate that exposure to reward contingencies within a semantic space, combined with gamification, stimulus spacing, or relaxed time constraints, can enable people to overcome an otherwise obligatory Pavlovian bias, so as to engage in optimal instrumental learning. Understanding the significance of the semantic response domain might be an essential next step in developing strategies that can help people suppress maladaptive behaviours, such as those seen in addiction.

## Methods

All participants were recruited from the Institute of Cognitive Neuroscience mailing list at University College London. Informed consent was obtained. Ethical approval for this study was obtained from the University College London Research Ethics Committee, application number 9929/002. All experiments were performed in accordance with the relevant guidelines and regulations. Stimuli were prepared in Inkscape. Tasks were coded in MATLAB with Cogent 2000 and Cogent Graphics.

### Massed motoric go/no-go task (MM)

In experiment 1 we recruited 42 participants (28 female) with a mean age of 24.2. These participants played a valenced go/no-go task 3 days in a row (Fig. 2A). Participants were instructed that they would encounter multiple abstract stimuli. For each stimulus, they had to learn whether it was better to ‘go’ (press a keyboard button) or ‘no go’ (do not press). In the ‘win’ conditions, a correct choice yielded points stochastically, with a probability $P$ and yielded no points with a probability of ($1-P$), whilst an incorrect choice yielded no points with a probability $P$ and yielded points with a probability of ($1-P$). In the ‘avoid losing’ conditions, a correct choice yielded no points with probability $P$ or resulted in losing points with probability ($1-P$), whilst an incorrect choice resulted in losing points with probability $P$, or yielded neutral feedback with probability ($1-P$). Participants were instructed to make as many correct choices as possible and that at the end of the 3 days their points would be converted into money that they could take home.

$P$ was computed as $(\mathrm{log}\left(t+1\right)/10)+0.5$ where $t$ was the trial number within the miniblock. Therefore, on the first trial of a miniblock, where $t=1$, $P$ was 0.57. On the 15th trial of a miniblock, where $t=15$, $P$ was 0.78. In this way, feedback became less stochastic and more deterministic throughout the duration of a miniblock. By making the feedback more deterministic at the end of each miniblock, we hoped to encourage subjects to learn each condition, and successfully experience the optimal instrumental strategy.

The stimulus was presented as a contextual cue in all four corners of the screen for the duration of a mini-block. Each mini-block lasted between 10 and 15 trials, the exact number selected at random from a uniform distribution. The variation in mini-block length ensured that participants could not predict when a mini-block would end and transition into a new one. Each trial started with a target circle randomly presented on either the left or right side of the screen. Subjects had 1000 ms to decide to ‘go’ or ‘no go’. To ‘go’, subjects had to press the arrow key corresponding to the side of the screen that the circle was on. To ‘no go’, subject had to press nothing at all. After making their choice, an outcome was shown for 900 ms. A green arrow indicated that points were won. A red arrow indicated that points were lost. A yellow bar indicated that points were neither won nor lost. There was then fixed inter-trial intervial (ITI) of 750 ms before the start of the next trial.

The task consisted of 44 mini-blocks, presented in a random order, each with a different abstract fractal stimulus. Therefore, 44 abstract fractal stimuli were encountered in total, 11 for each of the four conditions. Because the order of miniblocks was randomised, it was not possible for subjects to infer the condition of a miniblock from what they experienced in previous miniblocks. On each of the 3 days, a new set of 44 stimuli was encountered. On average, the task consisted of 550 trials, split evenly across the four conditions.

This behavioural task presented stimuli in a massed fashion, rather than a spaced fashion. In other words, within a miniblock, the same stimulus was presented repeatedly, rather than being interspersed with other stimuli. We therefore refer to this task as a massed, motoric go/no-go task (MM). The massed design removes most of the working memory demand imposed by typical valenced go/no-go paradigms, in which multiple stimuli need to be kept in working memory.

Initially, experiment 1 was intended to be a standalone experiment. As the project evolved, follow-up experiments were developed. Due to time constraints, these follow-up experiments had smaller sample sizes than experiment 1, with the target of at least 20 participants in each follow-up experiment.

### Spaced semantic go/no-go task with gamification (SS^g+^)

22 of the 42 participants played an additional task on the 3rd day of experiment 1 (Fig. 2B), after the final training session. This task was also a go/no-go task, but had a cover story. Subjects played the role of a sheepdog herding virtual sheep. Each sheep represented one of the four conditions of the valenced go/no-go task. Whenever the participant encountered a new cartoon sheep, they had to decide whether chase (go) or ignore (no-go) the sheep. Sometimes sheep responded by running into a barn (positive feedback). Sometimes sheep responded by running to the other side of the field (neutral feedback). Sometimes sheep responded by escaping the farm (negative feedback). Participants were instructed to learn which sheep they should chase and which sheep they should ignore, to herd as many sheep into the barn as possible, and stop as many sheep escaping as possible. They were instructed that their points would be converted into real money at the end of the experiment.

The task consisted of three blocks. Each block contained 15 trials with four different sheep. Therefore, a block contained 60 trials in total. These four sheep represented the four different conditions. The 60 trials were ordered randomly, so that subjects had to learn about all four sheep over the course of the block. At the end of the block, subjects were instructed that they were moving to a ‘new farm’ and so would now have to learn about a new set of four sheep.

Each trial started with a brief animation of a sheep jumping into the display and a sheepdog avatar appearing at the corner of the screen. Subjects had an unlimited amount of time to select the word ‘chase’ or ‘ignore’. On each trial, these words were randomly positioned such that one was on the left of the screen and one was on the right of the screen. If subjects chose ‘chase’ then the dog avatar moved towards the sheep. If subjects chose ‘ignore’ then the dog avatar moved away from the sheep, off the display. The outcome was then displayed, which showed an animation of either the sheep moving into the barn, the sheep moving to the other side of the field, or the sheep escaping the farm. Finally, there was a variable ITI of 1000–2000 ms before the start of the next trial. $P$ was fixed at 75% throughout the task.

This behavioural task presented stimuli in a spaced fashion. That is to say that within a block, a stimulus was re-encountered multiple times with other stimuli interspersed between trials. Furthermore, the response domain was semantic rather than motoric as the choice to ‘go’ or ‘no-go’ required identical motoric responses. We therefore refer to this task as a spaced, semantic go/no-go task with gamification (SS^g+^).

In experiment 2, a new sample of 26 participants (16 female) with a mean age of 22.7 was recruited and trained on SS^g+^ for 3 days in a row, with a new set of stimuli each day, and then also tested on MM on the 3rd day, after the final SS^g+^ session.

This experiment was initially conducted with a preliminary pilot sample of 14 participants. The data were analysed and a training effect was observed. At a later point in time, a secondary dataset of 12 participants was collected. The same training effect was observed in this second dataset. Here we report the results from these two datasets by pooling them into a single analysis of 26 participants.

### Spaced motoric go/no-go task without gamification (M^g−^)

In experiment 3 we recruited a new sample of 20 participants (15 female) with a mean age of 25.6. These subjects were trained on a new task for 3 days in a row (Fig. 4A), before being tested on MM on the 3rd day, after the final training session. This task was structured the same as SS^g+^, with three blocks of 60 trials. Each block contained 15 trials for each of the four conditions. However, as in MM, subjects gave responses in the motoric domain, by pressing or not pressing a button. Furthermore, stimuli were abstract fractal stimuli. There was no cover story. Subjects were instructed to win as many points as possible and that their points would be converted into real money at the end of the experiment. Subjects were told that they would encounter a new set of stimuli each day.

Within a trial, a fractal stimulus was presented, in the centre of screen, for a variable duration of 1000–2000 ms. A circle target was then presented on either the left or right side of the screen and subjects had up to 1000 ms to either press or not press. The outcome (green arrow, red arrow or yellow bar) was then presented for 1000 ms. Finally, there was a variable ITI of 1000–2000 ms before the start of the next trial. This trial structure was based on the original valenced go/no-go task design^21^. $P$ was fixed at 75% throughout the task. This behavioural task presented stimuli in a spaced fashion, but in a motoric domain. We therefore refer to this task as the spaced, motoric go/no-go task without gamification (M^g−^).

### Spaced motoric go/no-go task with gamification (M^g+^)

In experiment 4, we recruited a new sample of 18 participants (ten female) with a mean age of 27.3. These subjects were trained on a new task for 3 days in a row (Fig. 4B), before being tested on MM on the 3rd day, after the final training session. This task was almost identical to SS^g+^. Subjects were choosing to chase or ignore a sheep, but instead of selecting the word ‘chase’ or ‘ignore’, subjects had 1000 ms to choose to press a button or not a press a button, after seeing a circle target on either the left or right of the screen. The animations were identical to those in SS^g+^. $P$ was fixed at 75% throughout the task. Subjects were instructed to win as many points as possible and that their points would be converted into real money at the end of the experiment. Subjects were instructed that they would be encountering a new set of stimuli each day. We refer to this task as the spaced, motoric go/no-go task with gamification (M^g+^).

### Spaced semantic go/no-go task without gamification (SS^g−^)

In experiment 5 we recruited a new sample of 20 participants (11 female) with a mean age of 26. These subjects were trained on a new task for 3 days in a row (Fig. 4C), before being tested on MM on the 3rd day, after the final training session. This task was almost identical to M^g−^ but with responses given in the semantic domain rather than the motoric domain, with 1000 ms to choose to ‘chase’ or ‘ignore’.

Subjects were briefed with the same sheep cover story as the other semantic tasks, and were instructed that each sheep was labelled with a unique abstract fractal pattern. They were instructed that on each trial they would see the pattern of the sheep, and therefore have to decide whether to chase or ignore it. Subjects were instructed to win as many points as possible and that their points would be converted into real money at the end of the experiment. Subjects were instructed that they would be encountering a new set of stimuli each day. We refer to this task as the spaced, semantic go/no-go task without gamification (SS^g−^).

### Data analysis

For each experiment, we fit a single mixed logistic regression model to predict the binary choice of each individual trial as correct or incorrect, across the 3 days of training, using the fitglme function in MATLAB. Choice (go or no go), valence (win or avoid losing) and training session (day 1, day 2 or day 3), and all interactions between these three predictors, were included as both fixed effects and random effects, grouped by subject. We then tested the significance of each fixed effect term in the mixed model by performing an F test, testing whether all coefficients representing the fixed effect term were zero. A non-zero two-way interaction term between action and valence was taken as evidence for a Pavlovian bias. A non-zero three-way interaction term between action, valence and session was taken as evidence for a change in Pavlovian bias across the 3 days.

We also ran the same analysis within each individual training session (days 1, 2 and 3) and the final testing session on day 3, to investigate the strength of Pavlovian bias in each individual session.

Wherever we observed a null result, we conducted a Bayesian analysis in JASP^54^ to assess the strength of evidence in favour of the null hypothesis (e.g. absence of Pavlovian bias). In these analyses, we computed the proportion of correct responses for each subject, for each of the four conditions, and ran a Bayesian repeated measures ANOVA on these summary data. We used the default settings in JASP, including a uniform prior over models, and prior effects modelled as Cauchy distributions, centred on zero with an r scale of 0.5. We computed Bayes factors to assess the evidence in favour of the null hypothesis. For instance, a BF of 7 (log(BF) of 1.95) indicates that the posterior probability that the null model generated the data is seven times higher than the posterior probability that the alternative model generated the data.

## Supplementary Information

Supplementary Tables.
