# Supplementary material for: Overcoming Pavlovian bias in semantic space
Source: Sci Rep. 2021 Feb 9;11:3416. doi: 10.1038/s41598-021-82889-8 (PMC7873193; doi:10.1038/s41598-021-82889-8)
Supplement: Supplementary file 1 — Supplementary Tables. [file 41598_2021_82889_MOESM1_ESM.pdf]

Supplementary information for:

Overcoming Pavlovian bias in semantic space

Sam Ereira[1,2,*], Marine Pujol[1,3], Marc Guitart-Masip[1,4], Raymond J. Dolan[1,2], Zeb Kurth-Nelson[1,5]

[1]Max Planck UCL Centre for Computational Psychiatry and Ageing Research, UCL, London, WC1B 5EH, UK
[2]Wellcome Centre for Human Neuroimaging, UCL, London, WC1N 3BG, UK
[3]Sorbonne Université, Paris, France
[4]Aging Research Centre, Karolinska Institute, 171 65 Stockholm, Sweden
[5]DeepMind, London, N1C 4AG, UK
[*] e-mail: samuel.ereira.14@ucl.ac.uk

EFFECT	F	DF1	DF2	P
Intercept	2.17	1	68972	0.141
Choice	3.57	1	68972	0.056
Valence	12.48	1	68972	4.11×10^{-4}
Session	6.98	2	68972	9.33×10^{-4}
Choice*Valence	10.31	1	68972	0.0013
Choice*Session	6.38	2	68972	0.0017
Valence*Session	1.12	2	68972	0.326
Choice*Valence*Session	0.33	2	68972	0.72

Supplementary Table 1. Effect sizes from mixed logistic regression model in Experiment 1.

EFFECT	F	DF1	DF2	P
Intercept	46.67	1	14028	8.73×10^{-12}
Choice	11.41	1	14028	7.34×10^{-4}
Valence	11.93	1	14028	5.55×10^{-4}
Session	0.34	2	14028	0.709
Choice*Valence	43.6	1	14028	14.18×10^{-11}
Choice*Session	2.39	2	14028	0.092
Valence*Session	6.24	2	14028	0.002
Choice*Valence*Session	10.08	2	14028	4.23×10^{-5}

Supplementary Table 2. Effect sizes from mixed logistic regression model in Experiment 2.

EFFECT	F	DF1	DF2	P
Intercept	3.64	1	10788	0.056
Choice	18.99	1	10788	1.33×10^{-5}
Valence	44.49	1	10788	2.67×10^{-11}
Session	0.89	2	10788	0.412
Choice*Valence	31.72	1	10788	1.83×10^{-8}
Choice*Session	0.94	2	10788	0.392
Valence*Session	0.35	2	10788	0.706
Choice*Valence*Session	0.11	2	10788	0.895

Supplementary Table 3. Effect sizes from mixed logistic regression model in Experiment 3.

EFFECT	F	DF1	DF2	P
Intercept	11.37	1	9708	7.5×10^{-4}
Choice	0.033	1	9708	0.857
Valence	5.46	1	9708	0.0195
Session	12.78	2	9708	2.85×10^{-6}
Choice*Valence	12.1	1	9708	5.06×10^{-4}
Choice*Session	0.985	2	9708	0.374
Valence*Session	2.178	2	9708	0.113
Choice*Valence*Session	0.349	2	9708	0.705

Supplementary Table 4. Effect sizes from mixed logistic regression model in Experiment 4.

EFFECT	F	DF1	DF2	P
Intercept	19.34	1	10788	1.11×10^{-5}
Choice	0.0089	1	10788	0.925
Valence	6.02	1	10788	0.0142
Session	1.37	2	10788	0.255
Choice*Valence	9.24	1	10788	0.0024
Choice*Session	1.04	2	10788	0.354
Valence*Session	0.44	2	10788	0.644
Choice*Valence*Session	0.429	2	10788	0.651

Supplementary Table 5. Effect sizes from mixed logistic regression model in Experiment 5.
